# Long-term comparison of the efficacies of internal and external browpexy combined with blepharoplasty

**DOI:** 10.5935/0004-2749.20200033

**Published:** 2020

**Authors:** Ahmet Kaderli, Yasemin Katircioglu, Evin Singar Ozdemir, Sema Tamer Kaderli

**Affiliations:** 1 Ophthalmology Department, Mugla Sıtkı Koçman University, Turkey; 2 Ophthalmology Department, Ankara Training and Research Hospital, Ankara, Turkey

**Keywords:** Blepharoplasty, Eyebrow, Ptosis/surgery, Eyelids/surgery, Blefaroplastia, Sobrancelhas, Ptosis/cirurgia, Pálpebras/cirurgia

## Abstract

**Purpose:**

To perform a long-term comparison of the quantitative efficacy of internal and external browpexy in combination with upper-lid blepharoplasty based on lateral and central eyebrow positions.

**Methods:**

This retrospective study evaluated internal and external browpexy with upper-lid blepharoplasty surgeries that were performed during the period between January 2012 and December 2017 in the oculoplastic surgery department of our hospital. Patients who had undergone periorbital and forehead surgery, who had ophthalmologic or neurological diseases, and who were Botox users were not included in the study. Preoperative and postoperative measurements were made on photographs taken in the same position. The distances from the pupil center and from the point of intersection between the horizontal line passing through the pupil and the vertical line passing through the lateral canthus to the upper eyebrow borders were measured. Photogrammetric analysis of eyebrow position was analyzed using Corel Draw software.

**Results:**

Preoperative and postoperative photographs of 70 eyelids were analyzed. Measurements were taken 24 months after surgery. Mean elevations of 2.10 and 3.19 mm were observed in the central region and lateral regions, respectively, in the internal browpexy group. These elevations were 2.66 and 3.03 mm in the external browpexy group and 0.48 and 0.55 mm in the control group. Eyebrow elevations in the central and lateral regions were not significantly different from baseline in the control group (p=0.126 and p=0.25). Internal and external browpexy showed statistically similar elevation values in the central and lateral regions (p=0.636 and p=0.342).

**Conclusions:**

External and internal browpexy surgery afforded adequate and similar elevations of the central and lateral brow, which were significantly different from those in the standard blepharoplasty group during long-term follow-up.

## Abstract (pt)

**Objetivo:**

Avaliar a comparação, a longo prazo, da eficácia quantitativa da sobrancelha interna e externa em combinação com a blefaroplastia da pálpebra superior com base nas posições lateral e central da sobrancelha.

**Métodos:**

Este estudo retrospectivo avaliou as cirurgias interna e externa da braquiterapia com blefaroplastia de pálpebra superior rea lizadas entre janeiro de 2012 e dezembro de 2017 no setor de cirurgia Oculoplástica de nosso hospital. Pacientes submetidos à cirurgia periorbitária e frontal, com doenças oftal mológicas ou neurológicas e usuários de Botox não foram in cluídos no estudo. Medidas pré e pós-operatórias foram feitas em fotografias tiradas na mesma posição. Foram medidas as distâncias do centro da pupila e do ponto de intersecção entre a linha horizontal que passa pela pupila e a linha vertical que passa pelo canto lateral do olho até as bordas superiores da sobrancelha. A análise fotogramétrica da posição da sobrancelha foi analisada usando o software Corel Draw.

**Resultados:**

Foram analisadas fotografias pré e pós-operatórias de 70 pálpebras. As medidas foram feitas 24 meses após a cirurgia. Elevações médias de 2,10 e 3,19 mm foram observadas na região central e laterais, respectivamente, no grupo de sobrancelha interna. Essas elevações foram de 2,66 e 3,03 mm no grupo da sobrancelha externa e 0,48 e 0,55 mm no grupo controle (p=0,126, p=0,25). A sobrancelha interna e externa apresentou valores de elevação estatisticamente semelhantes nas regiões central e lateral (p=0,636, p=0,342).

**Conclusões:**

Cirurgia de sobrancelha externa e interna proporcionam elevações adequadas e semelhantes nas sobrancelhas central e lateral que foram significativamente diferentes daqueles no grupo de blefaroplastia padrão no acompanhamento a longo prazo.

## INTRODUCTION

Dermatochalasis, upper eyelid ptosis, and brow ptosis are common disorders of the aging process. Remodeling of the orbital rim due to lipoatrophy and increased skin laxity, which is a characteristic feature of aging ocu lar adnexal tissue, results in the inferior descent of the brows^(1)^. The lateral half of the brow tends to descend more than the medial half because the medial half exhibits more secure deep attachments^(2-5)^.

Lifting of the brow occasionally is combined with upper-lid blepharoplasty. Many procedures are sufficient to prevent brow descent following upper blepharoplasty^(6)^. These include internal and external brow lifting techniques. Browpexy is a simple process that is easily mastered and produces desired results with a high degree of patient satisfaction. Owing to the absence of an additional external incision, internal browpexy is the preferred technique. The advantage of internal brow lift procedures is that patients do not require secondary surgery. In addition, there is no visible scarring, as observed in direct brow lift, and there is no requirement for general anesthesia or deep sedation that is necessary in endoscopic brow lift^(7-10)^. External browpexy is a new surgical procedure that serves as an alternative to the internal browpexy and can be used to enhance upper blepharoplasty outcomes. This procedure suspends the brow through a small incision just within or above the upper brow cilia. As this technique requires a cutaneous incision, it generally heals without a perceptible scar. Another advantage of this procedure is that it is rapid, safe, and simple^(7,11)^.

Thus far, there is minimal evidence useful for the comparison of browpexy procedures in terms of long-term follow-up. In this study, we aimed to compare the long-term effects of internal and external browpexy combined with standard blepharoplasty.

## METHODS

### Patients

This was a retrospective review of 70 eyelids of 35 patients who were referred to the Oculoplastic Surgery Department of Ankara Research and Training Hospital during the period from January 2012 to December 2017. Internal and external browpexy with upper-lid blepharoplasty surgeries were performed for patients who had lateral brow ptosis complaints. The patients were classified into two groups: Group 1 included nine patients (18 eyelids) who underwent upper-lid blepharoplasty with internal browpexy, and group 2 included 12 patients (24 eyelids) who underwent upper-lid blepharoplasty with external browpexy. The control group comprised 28 eyelids of 14 patients who had only upper-lid blepharo plasty. The study was approved by the Institutional Review Board of Ankara Research and Training Hospital, and the review was performed in accordance with the tenets of the Declaration of Helsinki. Exclusion criteria included medial brow ptosis, prior surgery involving the eyebrow or eyelids, ocular trauma history, concomitant ptosis surgery, neuromuscular junction diseases, thyroid eye disease, and inadequate or missing photographs during follow-up.

**Figure 1:**
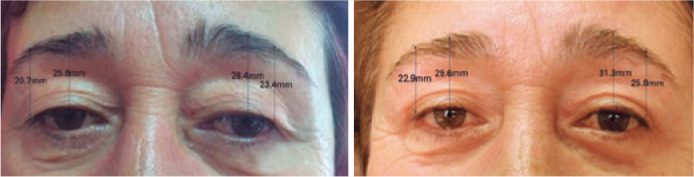
Preoperative and 24 months postoperative assessments of a patient who underwent combined internal browpexy and blepharoplasty surgery.

**Figure 2:**
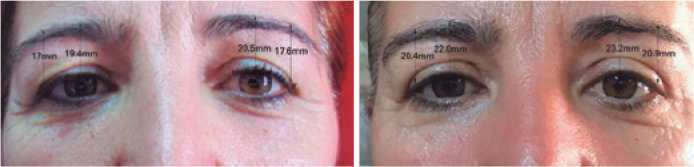
Preoperative and 24 months postoperative assessments of a patient who underwent combined external browpexy and blepharoplasty surgery.

**Figure 3:**
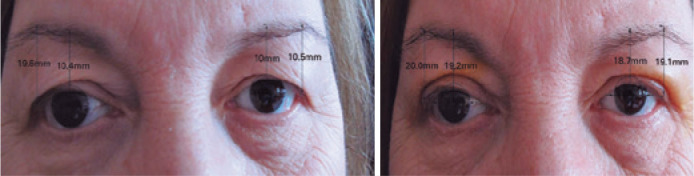
Preoperative and 24 months postoperative assessments of a pa tient who underwent a standard blepharoplasty surgery.

Preoperative and postoperative photographs of all patients were taken in the same position, and measurements were made using those photographs. All measurements included in the study were performed at 24 months postoperatively. Each photograph was taken by the same photographer, under the same lighting conditions, at a fixed distance between the patient and the camera. A 20.1-megapixel Canon PowerShot G7X Digital ca mera was used in a standard manner. The central region was defined as the vertical range between the pupil center and the upper eyebrow border. The lateral region was defined as the vertical range between the point of intersection of the horizontal line passing through the pupil and the vertical line passing through the lateral cantus to the upper eyebrow border (Figures 1-3). Photogrammetric analysis of eyebrow position was performed using Corel Draw software. The software standardized measurements by fixing the corneal diameter at 10 mm.

### Surgical technique

All surgeries were performed by the same surgeon (Y.K.)

**Internal browpexy:** Fixation of the brow to the supraorbital rim periosteum can provide elevation of the ptotic or lax brow. The desired location of the lateral brow is typically set by elevating the inferior brow to a level 1 cm above the superior orbital rim. Prior to the operation, the brow fixation point was marked at a point connecting the nasal ala and mid-pupillary line, with patients in the sitting position. Upper blepharoplasty was performed with skin removal. A 4-0 polypropylene (Prolene) suture was passed transcutaneously from the lower edge of the brow hairs into the previously dissected sub-brow space. The same suture was then sewn through the remaining sub-brow and periosteum above the orbital rim. The suture was then passed again into the sub-brow muscular tissue at the level of the original transcutaneously passed marking suture. The transcutaneous suture was removed, and the suture was tied using a loop over. Care was taken to avoid over-tightening the suture when adequate placement is achieved, as this may immobilize the brow.

**External browpexy:** Prior to the operation, the desired height of elevation was determined with patients in the sitting position. The brow fixation point was marked at a junction of the middle and outer thirds of the brow point. An 8-mm demarcation was made preoperatively, superior to the upper brow cilia. In this predetermined area, an incision through the dermis was made by Westcott scissors following the curvature of the brow. The incision was extended through the orbicularis muscle and the brow fat pad to the level of the periosteum of the frontal bone. The periosteum was engaged with forceps and dissected to create a dissection pocket with a horizontal dimension of approximately 15 mm. This allowed a small skin entry point (minimally visible incision) and a larger internal dissection and resultant scar to aid in the maintenance of the brow position after the operation. The periosteum at this level was engaged with a 4-0 Prolene suture and was secured to the orbicularis muscle and brow fat pad at the inferior lip of the incision in a pseudo-mattress manner. The subcutaneous tissue and skin were closed with interrupted 6-0 vicryl sutures.

**Blepharoplasty:** Preoperative markings were made with the patient sitting upright in neutral gaze with the brow properly positioned. The eyelid crease was situated above the ciliary margin approximately 8-9 mm in women and 7-8 mm in men. The extent of excision was at least 10 mm from the inferior border of the brow. The upper lids were injected superficially with 2% lidocaine containing 1:100,000 epinephrine using a 27-30-gage needle. A skin incision was made with an Empire tip by radiofrequency monopolar cautery. No orbicularis muscle excision was done. Three absorbable sutures were used to incorporate the orbicularis from the lower and upper edges of the incision along with the superolateral arcus marginalis to form the upper-lid crease. The skin incision was closed using running absorbable 6-0 vicryl sutures.

### Statistical analysis

Statistical analysis was performed using SPSS for Windows software (version 14.01; SPSS, Chicago, IL, USA). All values are presented as the mean ± standard error of mean. The Shapiro-Wilk test was used to assess whether quantitative variables exhibited a normal distribution, and homogeneity of variance was examined using the Levene test. The general linear modeling method was used for repeated measurements to examine temporal differences between central and lateral region measurements obtained before and after the operation based on the methods used. The included model included Time (Preop and Postop), Group (1, 2, and 3), and Time*Group interaction terms. For significant interaction terms, simple effects analysis was performed with post hoc Bonferroni correction p<0.05 was considered statistically significant unless otherwise noted.

## RESULTS

Patients’ ages ranged from 55 to 74 years (mean 64.97 ± 0.82); 27 (77.1%) patients were women, and eight patients (22.9%) were men. The mean follow-up time was 33.3 ± 0.61 months. The demographic characteristics of the patients are shown in table 1. The in ternal browpexy group included seven women and two men with an average age of 61.3 years, the external browpexy group included nine women and three men with an average age of 65.5 years, and the control group included 11 women and three men with an average age of 66.7 years. No significant differences were found among the three groups according to age, sex, or follow-up period (p>0.05).

**Table 1:** Demographic data of each group

	Blepharoplasty (control)		Internal browpexy		External browpexy
Total number of eyelids	28		18		24
Mean age (years)	66.7 ± 1.09		61.3 ± 1.6		65.5 ± 1.3
Mean follow-up (months) ± SEM	34.6 ± 1.0		34.5 ± 1.4		30.9 ± 0.7
Women/Men	11 (78.6%)/3 (21.4%)		7 (77.8%)/2 (12.2%)		9 (75%)/3 (25%)

SEM= standard error of the mean.

Mean elevations of 2.10 and 3.19 mm were observed in the central region and lateral regions, respectively, in the internal browpexy group. These elevations were 2.66 and 3.03 mm in the external browpexy group and 0.48 and 0.55 mm in the control group. There was a statistically significant difference between preoperative and postoperative measurements in the internal and external browpexy groups (p<0.001). Eyebrow elevations in the central and lateral regions were not statistically significant in the control group (p=0.126 and p=0.25).

Internal and external browpexy showed statistically similar elevation values in the central and lateral regions (p=0.636 and p=0.342). Both methods provided a significant increase in both areas, compared with that provided by a standard blepharoplasty surgery (p<0.05; table 2).

**Table 2:** Comparison of postoperative mean elevations among groups

Group	n	Mean elevation in the central region (mm) ± SEM		Mean elevation in the lateral region (mm) ± SEM	
Internal browpexy^a^	18	2.10 ± 0.34		3.19 ± 0.59	
External browpexy^a^	24	2.66 ± 0.3		3.03 ± 0.51	
Blepharoplasty (control)^b^	28	0.48 ± 0.27		0.55 ± 0.47	

**P values for the central region:** Time <0.001; Group= 0.636; Time^*^Group <0.001 **P values for the lateral region:** Time <0.001; Group= 0.342; Time^*^Group <0.001 a,b= Different letters in the same column indicate statistically significant differences (p<0.001).

SEM= standard error of the mean.

There were no unacceptable scars or facial nerve injuries in any patient, and none of the patients complained of eyebrow loss.

## DISCUSSION

This study showed that both browpexy techniques produced statistically significant elevations in central and lateral brow heights. External and internal browpexy surgeries provided similar elevations of the central and lateral brow, and both techniques achieved adequate brow lift. When compared to standard blepharoplasty (control) surgery, the amount of lift for both procedures was statistically significant.

Brow ptosis is a common cosmetic and functional diagnosis in elderly patients. Decisions regarding brow lifting surgical technique should consider preoperative brow position, height, contour, and the patient’s expectations^(6)^. Although direct and endoscopic brow lifting procedures show significantly greater brow elevation and provide excellent results, they are costly and invasive and can lead to significant complications such as scarring, alopecia, and nerve injury^(6,10)^. These techniques are complicated surgeries with longer recovery times, so it is crucial for surgeons to assess the position of the brow and choose the appropriate surgical technique to ensure the best cosmetic and functional outcomes. Mokhtarzadeh et al. reported that external and internal browpexy surgeries produced similar elevations of the central and lateral brow at 4-5 months postoperatively^(12)^. Our study results showed higher elevation values in the central and lateral regions in long-term follow-up after both procedures.

There are many contradictory reports regarding brow position after blepharoplasty without browpexy surgery. Faigen reported reductions of both central and lateral brow after blepharoplasty if either browpexy technique was not used^(13)^. In our study, standard blepharoplasty surgery supported brow lift, but the resulting elevation was not statistically significant. Therefore, to avoid brow ptosis after blepharoplasty surgery, we recommend the use of either browpexy method. Prado et al. also reported brow lift after upper blepharoplasty surgery, and they found that, because the lateral portion of the eyebrow is not strongly adhered to the subjacent structures, alterations may occur in the eyebrow positioning^(14)^. They also recommended correction of the eyebrow position along with blepharoplasty to avoid alterations in the eyebrow position in individuals with flaccid tissues in the lateral portion^(14)^.

Each browpexy technique has unique advantages and disadvantages. The direct brow lift can provide adequate lift but can change brow shape, produce an inconvenient scar, and/or cause neurological problems such as motor paresis or sensory deficit^(10)^. Internal browpexy produces no serious scarring on the skin, but outcomes can be unpredictable^(11)^. Eternal browpexy is rapid and minimally invasive, but scarring may be serious^(11)^.

The limitations of this study include its lack of medial brow height evaluation and a predominance of women among the included patients. The medial brow position was not considered because lateral brow procedures were not assumed to affect the medial side of the brows. The predominance of women is associated with an in creased preference for this type of surgery among women. In addition, this was a retrospective study, and given that patients were not randomized to the various procedures, there was a degree of selection bias.

In summary, this study showed that external and internal browpexy surgeries provide similar and statistically significant elevations in central and lateral brow heights. The standard blepharoplasty technique also provided eyebrow elevations in the central and lateral regions, but these elevations were not statistically significant. To the best of our knowledge, this is the first series to compare long-term quantitative outcomes between internal and external procedures.
